# Rebaseline no evidence of disease activity (NEDA-3) as a predictor of long-term disease course in a Norwegian multiple sclerosis population

**DOI:** 10.3389/fneur.2022.1034056

**Published:** 2022-11-14

**Authors:** Cecilia Smith Simonsen, Heidi Øyen Flemmen, Line Broch, Kamilla Brekke, Cathrine Brunborg, Pål Berg-Hansen, Elisabeth Gulowsen Celius

**Affiliations:** ^1^Department of Neurology, Vestre Viken Hospital Trust, Drammen, Norway; ^2^Department of Neurology, Hospital Telemark HF, Skien, Norway; ^3^Institute of Clinical Medicine, University of Oslo, Oslo, Norway; ^4^Department of Neurology, Oslo University Hospital, Oslo, Norway; ^5^Department of Neurology, Hospital Vestfold, Tønsberg, Norway; ^6^Oslo Centre for Biostatistics and Epidemiology, Research Support Services, Oslo University Hospital, Oslo, Norway

**Keywords:** multiple sclerosis, NEDA 3, no evidence of disease activity, time to EDSS 6, high efficacy treatment

## Abstract

**Introduction:**

No evidence of disease activity with three components (NEDA-3) is achieved if the person with MS (pwMS) has no new MRI lesions, no new relapses and no change in Expanded disability status scale (EDSS) over 1 year. Whether NEDA-3 is a good tool in measuring disease activity is up for discussion, but it is superior to the individual parameters separately and user-friendly. There is disagreement on whether NEDA-3 is a good predictor of long-term disability.

**Methods:**

This is a retrospective cohort study using real-world data with limited selection bias from the complete MS population at two hospitals in the southeast of Norway. We included pwMS diagnosed between 2006 and 2017 who had enough information to determine time to failure of NEDA-3 after diagnosis.

**Results:**

Of 536 pwMS, only 38% achieved NEDA 1 year after diagnosis. PwMS achieving NEDA were more likely to be started on a high efficacy drug as the initial drug, but there were no demographic differences. Mean time to NEDA failure was 3.3 (95% CI 2.9–3.7) years. Starting a high efficiacy therapy was associated with an increased risk of sustaining NEDA as compared to those receiving moderate efficacy therapy. PwMS who achieved NEDA at year one had a mean time to EDSS 6 of 33.8 (95% CI 30.9–36.8) years vs. 30.8 (95% CI 25.0–36.6) years in pwMS who did not achieve NEDA, p < 0.001. When rebaselining NEDA 1 year after diagnosis, 52.2% achieved NEDA in the 1st year after rebaseline, mean time to NEDA failure was 3.4 (95% CI 3.0–3.7) years and mean time to EDSS 6 was 44.5 (95% CI 40.4–48.5) years in pwMS achieving NEDA vs. 29.6 (95% CI 24.2–35.0) years in pwMS not achieving NEDA, *p* < 0.001. After rebaseline, pwMS with a high efficacy therapy as the initial drug had a mean time from diagnosis to NEDA fail of 4.8 years (95% CI 3.9–5.8) vs. 3.1 years (95% CI 2.7–3.5) in pwMS started on a moderate efficacy therapy, *p* < 0.001. In pwMS with NEDA failure at year one, 70% failed one, 28% failed two and 2% failed three components. New MRI lesions were the most common cause of NEDA failure (63%), followed by new relapses (50%) and EDSS change (25%).

**Conclusion:**

NEDA-3 from rebaseline after 1 year, once treatment is stabilized, can predict the long-term disease course in MS. Starting a high efficacy DMT is associated with longer time to NEDA failure than moderate therapies. Finally, most pwMS only fail one component and new MRI lesions are the most likely cause of NEDA failure.

## Introduction

The prognosis of multiple sclerosis (MS) varies greatly in individual people with MS (pwMS) from very mild to very serious, though the disease course has improved significantly over the past few decades. Much of this improvement, though not all, can be attributed to increasingly more potent disease modifying therapies (DMTs) (1, 2). Prognostic markers in MS are highly sought after and “No evidence of disease activity” (NEDA) early in the disease course has been flagged as a potential tool to predict long term disability. The classical NEDA, or NEDA-3, as it is often referred to, is defined as (1) no new or enlarging T2 weighted lesions or gadolinium enhancing lesions on MRI of the brain, (2) no new clinical relapses, and (3) no confirmed worsening of EDSS (3). There is disagreement on whether NEDA can be used as an early predictor of long-term disability, with some studies saying that it can (4–7) and others saying that it is unsuitable for this purpose (8–10). Critics of NEDA highlight its overreliance on MRI and lack of sensitivity in detecting degeneration and low-grade inflammation (11) as well as white matter microstructural deterioration (12), with many authors suggesting additional components to increase its accuracy (11, 13). Others, again, have concluded that minimal evidence of disease activity (MEDA), defined as ≤ 2 new MRI lesions but no progression in EDSS or relapses, may be tolerated without exposing the pwMS of future disability (14). Though NEDA is not a perfect tool, it does say something about disease activity in the first few years after treatment initiation, which in itself is an important prognostic factor (15, 16). In addition, it is a simple tool that is better than the three individual components separately (3) and it is easy to implement in routine clinical practice. We sought to determine whether NEDA can be used as a predictor for long term disability. In addition, we wanted to disentangle which components of NEDA are most likely to fail.

## Methods

The BOT-MS (Buskerud, Oslo and Telemark) is a database comprising the complete population of pwMS in the two counties of Buskerud and Telemark, as well as the majority of the pwMS in the Norwegian capital Oslo (*n* = 3,951). For this study we only included the complete populations from Buskerud and Telemark, as the Oslo population is not as complete as the two other counties and may introduce a selection bias. Data were recorded prospectively until 31.12.2017, but retrieved retrospectively by three neurologists specialized in MS between January and December 2018. Detailed information on the database and data collection has previously been published (1). For the current study, we only included pwMS diagnosed by January 2017 to ensure at least 1 year follow-up information.

**Figure 1:**
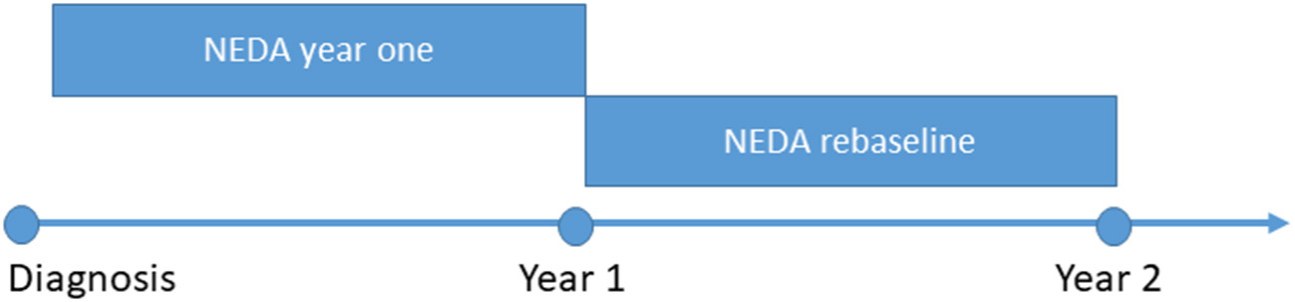
Definitions of NEDA used in this paper. NEDA no evidence of disease activity.

PwMS with missing or incomplete information and pwMS with incomplete information precluding determination of NEDA were excluded. New or enlarging lesions, or new gadolinium enhancing lesions on follow-up brain MRI were considered as MRI change. Any increase in EDSS on at least two consecutive occasions was deemed as a worsening of EDSS. We chose this definition of disease progression in order to capture any possible clinical activity due to treatment failure, though we are aware that this might reduce the number of pwMS reaching NEDA. Only EDSS documented 3 months or more after a relapse were included. Almost all EDSS changes were verified by a new examination after at least 6 months. Any relapses documented in the person's hospital records, regardless of steroid treatment, constituted a relapse. NEDA fail is defined as a positive finding in one or more components (EDSS, MRI and/or relapse), even in cases where we were missing one or two of the other components. We have looked at NEDA/NEDA fail at two time points (Figure 1). “NEDA at year one” was determined 1 year after diagnosis. “NEDA rebaseline” was determined using 1 year after diagnosis as the new baseline. When using pwMS with NEDA rebaselined, we excluded pwMS who had a known NEDA status in year one, but not in year two. MEDA was defined as a low MAGNIMS score (17), with zero, one or two new MRI lesions, no contrast enhancement; no new relapses or worsening in EDSS (Prosperini et al.). Consequently, MEDA includes all pwMS with NEDA as well as pwMS with one or two new lesions, but otherwise stable disease. Time to NEDA fail was defined as years from diagnosis to the year the pwMS failed NEDA. Time to EDSS 6 was defined as years from onset to when the pwMS became dependent on intermittent or unilateral walking aid to walk 100 meters (18). Follow-up time was calculated as years from time of onset until the date of NEDA fail/EDSS 6, date of emigration, death or to January 1st, 2018, whichever occurred first.

Only pwMS diagnosed in 2006 or after were included as this was the 1st year our population had access to the first high efficacy drug, natalizumab. Consequently, follow-up routines were more stringent as of 2006. In the analyses, we only included the first drug used. We considered interferons, glatiramer acetate, teriflunomide and dimethyl-fumarate as moderate efficacy disease modifying drugs (DMTs), and natalizumab, fingolimod and alemtuzumab as high efficacy DMTs. We only included drugs that had been used for at least 3 months and alemtuzumab was considered effective from the first treatment. All DMTs were available for all pwMS from market access in Europe.

### Statistics

We used IBM SPSS Statistics 25.0 (IBM Corp., Armonk, NY, USA) for data analysis. Between groups, differences in continuous variables were tested with Student *t*-test for normally distributed data and Mann-Whitney U-test for skewed data. The chi-square test for contingency tables was used to detect associations between categorical variables. To examine whether the proportion of patients who achieved NEDA increased by years of diagnosis, binary logistic regression analysis was used. In the trend analysis, the year/time factor/variable were treated as a ordinal score and we adjusted for the confounding effects of time to start of medication, type of medication and/or time from onset to diagnosis. There was no multicolinearity. We used the Kaplan-Meier method to calculate time to NEDA fail and EDSS 6, and Log-Rank test to compare groups. We also used cox regression analysis to calculate the hazard ratio of sustaining NEDA status and reaching EDSS 6, before and after adjusting for the confounding effects of time to start of medication, type of medication and/or time from onset to diagnosis, as well as gender and age at diagnosis. All *p*-values were two-sided and a 5% significance level was used.

### Ethics

The Regional Ethics Committee in Norway (REK 2015/670) approved this study under the condition that strict privacy concerns were respected, and that data was not made publicly available. Specific requests regarding data sharing should be directed to the corresponding author.

## Results

### Population

A total of 615 pwMS were diagnosed between January 2006 and January 2017 in the two counties of Buskerud and Telemark. We have enough information to determine NEDA in year one on 536 of these (87%). If only including those with at least 2 years follow-up, we have information on NEDA rebaseline on 446 pwMS (86.1% of pwMS with at least 2 years follow-up). See Table 1 for demographic information on the population as a whole and those included and missing. PwMS in Telemark were more likely to have sensory symptoms at onset than PwMS in Buskerud (41.7 vs. 32.1%, *p* = 0.024), and time from onset to diagnosis was shorter at 3.8 (SD 6.2) vs. 5.1 (SD 7.4), *p* = 0.043. Otherwise, there were no significant demographic differences between the two hospitals.

**Table 1:** Demographic information on the population as a whole and those included and missing.

	**All**	**Included in NEDA year one**	**Missing**	***p***
	**(*n* = 615)**	**(*n* = 536)**	**(*n* = 79)**	
Women, %	67.0	67.4	64.6	0.622
Mean age at onset, years (SD, range)	37.0 (11.6, 10–76)	36.6 (11.5, 10–76)	40.0 (11.9, 11–65)	0.018
Mean age at diagnosis, years (SD, range)	42.2 (12.5, 11–76)	41.2 (12.2, 11–76)	49.1 (12.6, 15–75)	< 0.001
Years onset to diagnosis, mean (SD, range)	5.2 (7.8, 0–54)	4.5 (6.9, 0–53)	9.7 (11.4, 0–54)	< 0.001
Progressive at onset, %	11.0	8.7	27.0	< 0.001
≥2 relapses before diagnosis, %	67.0	68.1	59.1	0.145
Sensory symptoms at onset, %	36.0	36.3	33.8	0.676
Motor symptoms at onset, %	20.7	19.0	3.4	0.008
EDSS at diagnosis, median (IQR)	2.5 (1.5,3.0)	2.5 (1.5,3.0)	2.5 (2.0,3.0)	0.322
Start DMT within 6 months of diagnosis, %	80.7	81.8	62.5	0.020
Initial drug is high efficacy therapy, %	18.7	18.7	18.5	0.979
Time to EDSS 6, years (95% CI)	36.1 (32.4–39.8)	35.4 (30.9–40.0)	36.0 (28.8–43.2)	0.672

SD, standard deviation; EDSS, expanded disability status scale; IQR, interquartile range; DMT, disease modifying therapy; NEDA, no evidence of disease activity; CI, confidence interval.

### No evidence of disease activity

**Figure 2:**
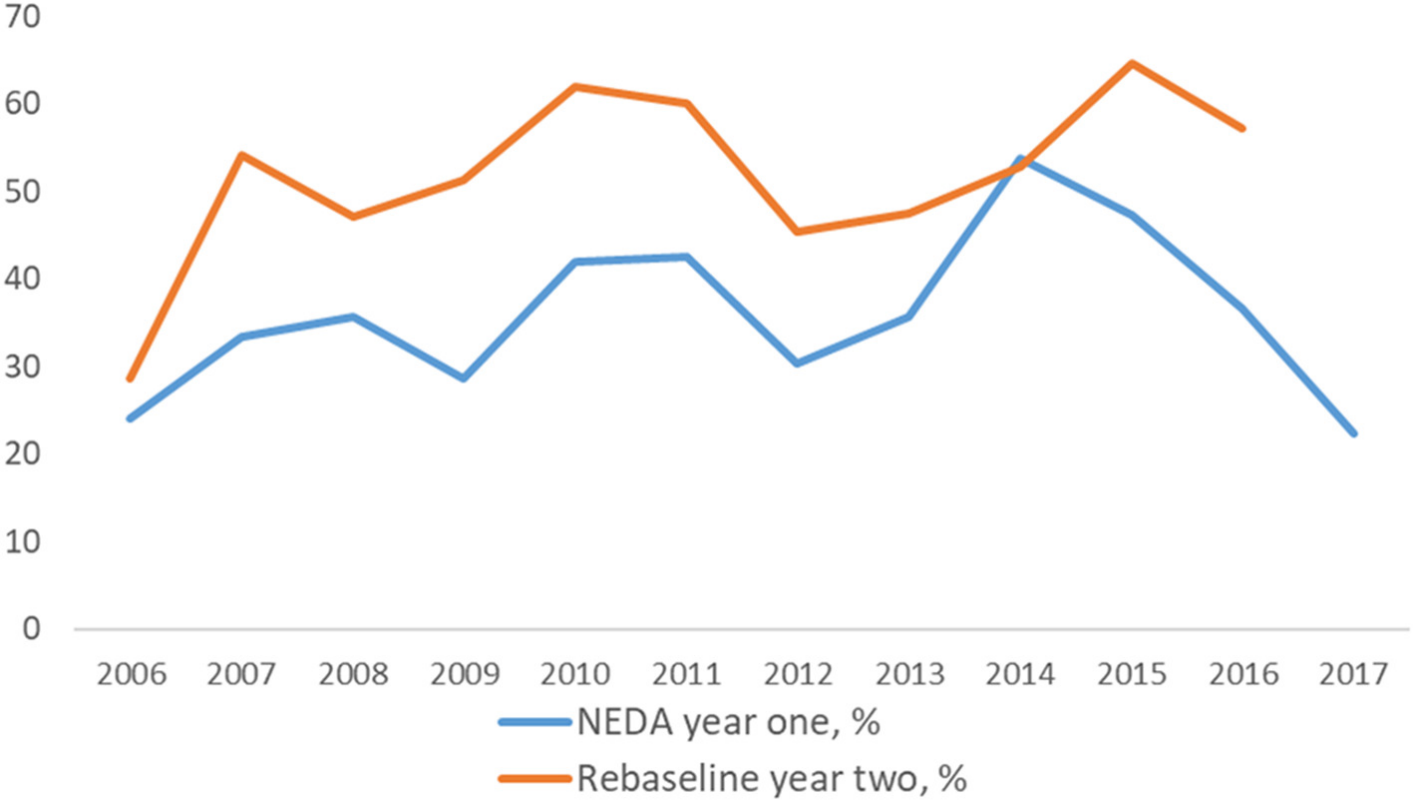
Proportion of pwMS who achieved NEDA at year one and at rebaseline by year of diagnosis. NEDA, no evidence of disease activity.

From the population with enough information to determine NEDA in year one, 38% achieved NEDA (*n* = 202) and 62% did not achieve NEDA at year one (*n* = 334). Of pwMS who did not achieve NEDA at year one, 35% of pwMS achieved NEDA at year two (*n* = 117), At year one, 47% of the population with enough information achieved MEDA and 53% (284) did not achieve MEDA. When rebaselining, 52% achieved NEDA (*n* = 233) and 48% did not achieve NEDA (*n* = 213). Figure 2 shows the proportion of pwMS who achieved NEDA at year one and at rebaseline. There was no significant association over time in the proportion of pwMS achieving NEDA in year one (Odds ratio (OR) per 1 year increase 1.049 (95% confidence interval (CI) 0.995–1.107), p_trend_ = 0.254). However, there was a significantly positive trend at rebaseline (OR 1.067 (95% CI 1.000–1.138), p_trend_ = 0.050), though significance vanished after adjusting for time to start of medication, type of medication and/or time from onset to diagnosis (OR 1.041 (95% CI 0.959–1.131), p_trend_ = 0.334). PwMS who achieved NEDA in year one and at rebaseline were more likely to have started disease modifying therapy (DMT) within 6 months of diagnosis. Apart from this, there were no significant differences in demographics and disease characteristics between pwMS who did and did not achieve NEDA at year one and when rebaselining, see Table 2. In year one, 33% of Buskerud pwMS and 44% of Telemark pwMS reached NEDA, *p* = 0.009. If rebaselining at year two, 45% of Buskerud and 63% of Telemark pwMS reached NEDA, *p* < 0.001.

**Table 2:** Disease characteristics and demographics in pwMS who did and did not achieve NEDA at year one and at rebaseline.

	**Year one**			**Rebaseline**		
	**NEDA**	**NEDA fail**	***p***	**NEDA**	**NEDA fail**	***p***
	**(*n* = 202)**	**(*n* = 334)**		**(*n* = 233)**	**(*n* = 213)**	
Women, %	67.3	67.4	0.993	69.5	68.7	0.853
Mean age at onset, years (SD, range)	36.7 (11.9, 15–64)	36.5 (11.2, 10–76)	0.845	36.1 (11.0, 15–63)	36.5 (11.3, 10–66)	0.704
Mean age at diagnosis, years (SD, range)	41.7 (12.4, 17–73)	40.9 (12.0, 11–76)	0.457	40.5 (11.4, 16–68)	41.6 (12.8, 11–75)	0.346
Years onset to diagnosis, mean (SD, range)	5.0 (7.2, 0–32)	4.3 (6.7, 0–53)	0.267	4.4 (6.6, 0–50)	5.0 (7.4, 0–41)	0.405
Progressive at onset, %	8.5	8.9	0.863	7.4	11.2	0.178
≥2 relapses before diagnosis, %	65.3	72.4	0.092	68.5	71.6	0.480
Sensory symptoms at onset, %	34.2	37.5	0.440	38.5	35.6	0.536
Motor symptoms at onset, %	18.4	19.4	0.774	16.8	19.0	0.550
EDSS at diagnosis, median (IQR)	2.0 (2.0, 3.0)	2.5 (2.0, 3.5)	0.737	2.5 (2.0, 3.0)	2.5 (2.0, 3.0)	0.284
DMT within 6 months of diagnosis, %	84.7	80.1	0.250	83.5	75.2	0.062
Initial drug is high efficacy therapy, %	26.4	14.3	0.003	20.1	10.3	0.014

SD, standard deviation; EDSS, expanded disability status scale; IQR, interquartile range; DMT, disease modifying therapy; NEDA, no evidence of disease activity; CI, confidence interval.

### Time to NEDA fail

**Figure 3:**
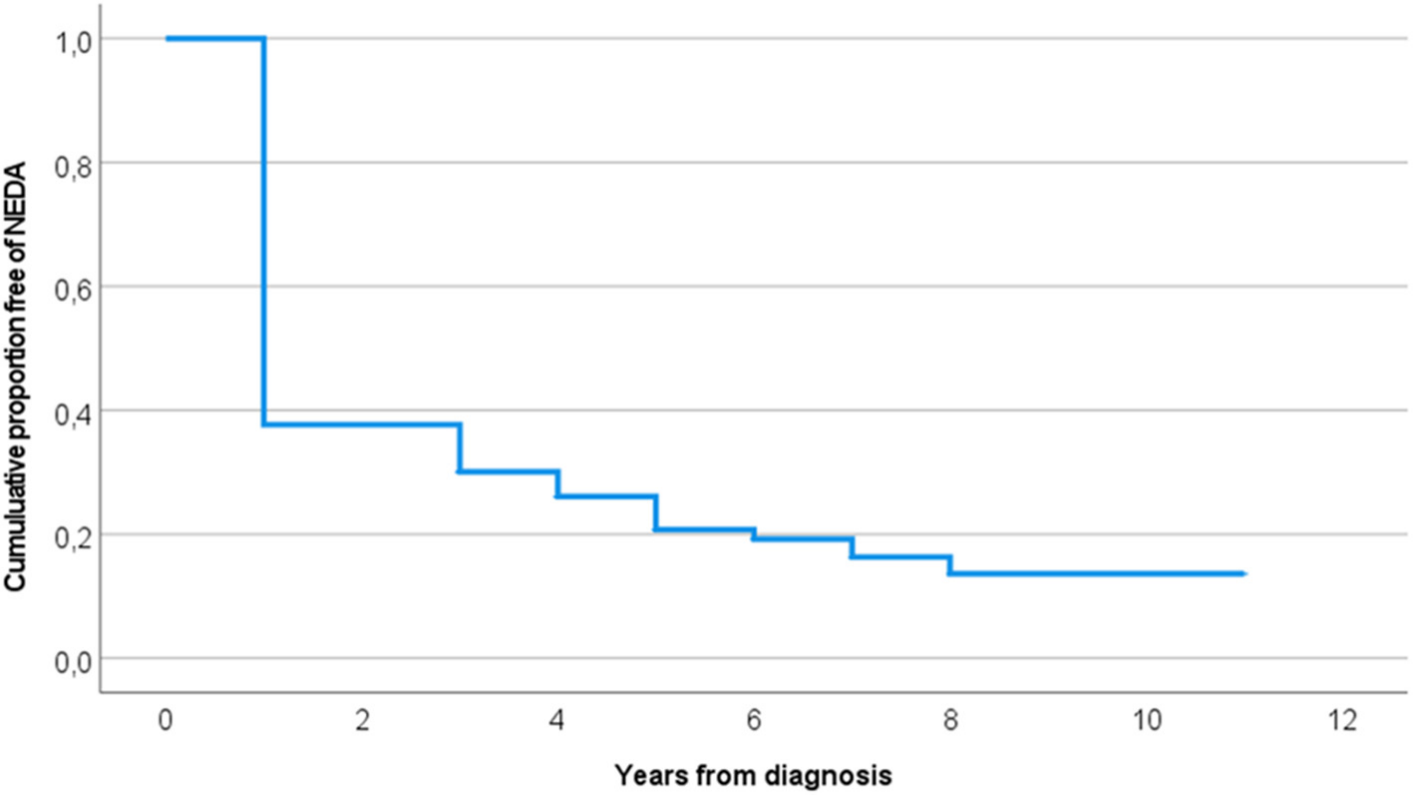
Time to NEDA fail in years. NEDA no evidence of disease activity.

The mean time to NEDA fail was 3.3 years (95% CI 2.9–3.7), see Figure 3. If rebaselining after one year, time to NEDA fail was 3.4 (95% CI 3.0–3.7) years. Mean time to NEDA fail in year one in pwMS with relapsing MS (*n* = 481) was 3.4 (95% CI 3.0–3.8) years and progressive MS (*n* = 46) was 2.7 (95% CI 1.8–3.5) years, *p* = 0.494.

### Achieving NEDA on moderate efficacy vs. high efficacy therapy

We divided the population into those who started on a high efficacy disease modifying therapy (DMT) (*n* = 76) and moderate efficacy DMT (*n* = 330) as the initial drug. Mean time to NEDA fail was 3.7 (95% CI 3.0–4.4) years in the high efficacy group and 2.8 (95% CI 2.4–3.2) years in the moderate efficacy group, 0 < 0.001. If rebaselining after 1 year, mean time to NEDA fail was 4.8 (95% CI 3.9–5.8) years in the high efficacy group and 3.1 (95% CI 2.7–3.5) years in the moderate efficacy group, *p* < 0.001. Median time to therapy initiation from diagnosis was within 1.0 month (IQR 0,2) for the high efficacy group and within 2.0 months (IQR 0,3) in the moderate efficacy group, *p* = 0.014.

**Figure 4:**
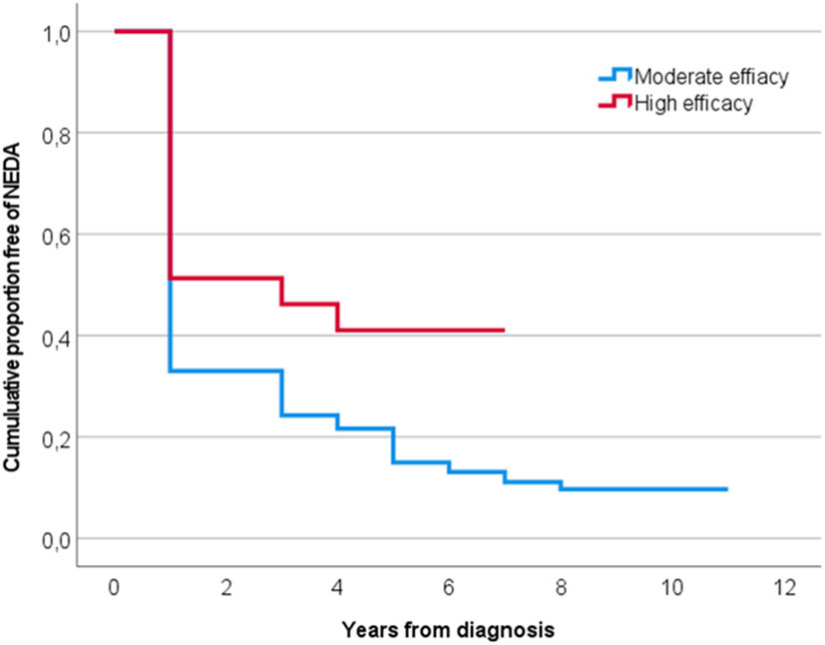
Years to NEDA fail in pwMS started on moderate (blue) and high (red) efficacy therapy within 6 months. NEDA no evidence of disease activity.

If only looking at pwMS started on a drug within 6 months of diagnosis, the mean time to NEDA fail in the high efficacy group (*n* = 64) was 4.0 (95% CI 3.2–4.8) years vs. 2.9 (95% CI 2.4–3.4) years in the moderate efficacy group (*n* = 258), *p* < 0.001, see Figure 4. When rebaselining, mean time to NEDA fail in pwMS started on a high efficacy therapy was 4.8 (95% CI 3.9–5.8) years and 3.3 (95% CI 2.9–3.8) years in pwMS started on a moderate efficacy therapy, p = 0.001.

Starting a high efficiacy therapy was associated with an increased risk of sustaining NEDA as compared to those receiving moderate efficacy therapy, also after adjusting for gender, age at diagnosis, time to start of medication, high or moderate efficacy therapy as initial DMT and time from onset to diagnosis (see Table 3).

**Table 3:** Hazard ratio of sustained NEDA on high efficacy therapy compared to moderate efficacy therapy at year one and at rebaseline, before and after adjustment using cox regression analysis.

**Year 1**	**HR of sustained NEDA**	**95% CI**		***p***
High efficacy first, crude	1.463	1.047	2.042	0.026
High efficacy first, adjusted	1.434	1.016	2.025	0.040
Female	0.928	0.721	1.193	0.559
Age at diagnosis	0.998	0.986	1.010	0.707
Years from onset to diagnosis	0.994	0.971	1.017	0.599
DMT within 6 months of diagnosis	0.857	0.643	1.140	0.289
**Rebaseline**				
High efficacy first, crude	1.803	1.173	2.772	0.007
High efficacy first, adjusted	1.710	1.106	2.646	0.016
Female	0.934	0.704	1.238	0.635
Age at diagnosis	0.999	0.986	1.013	0.929
Years from onset to diagnosis	0.991	0.965	1.018	*0.522*
DMT within 6 months of diagnosis	0.793	0.587	1.072	*0.131*

HR, hazard ratio; NEDA, no evidence of disease activity; DMT, disease modifying therapy; CI, confidence interval.

### Time to EDSS 6

**Figure 5:**
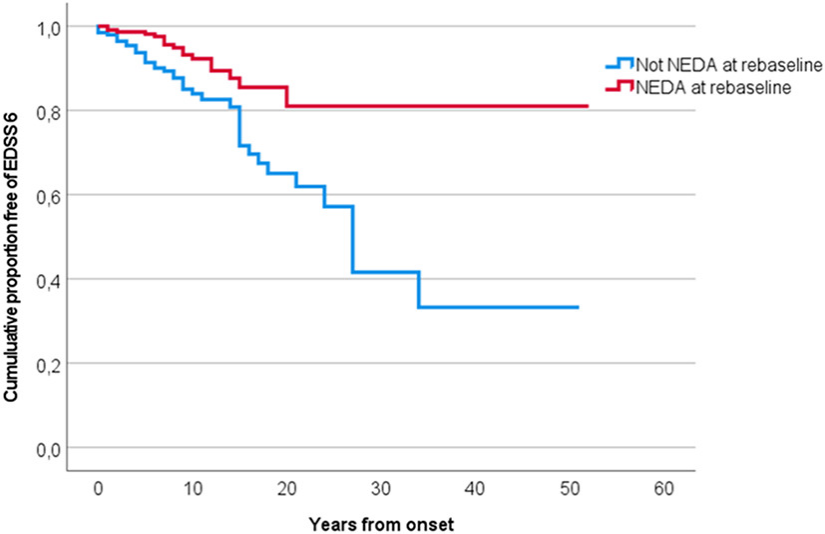
Time to EDSS 6 in pwMS who achieved NEDA (red) and pwMS who did not achieve NEDA (blue) when rebaselined.

PwMS who achieved NEDA at year one after diagnosis had a mean time to EDSS 6 of 33.8 years (95% CI 30.9–36.8) vs. 30.8 years (95% CI 25.0–36.6) in pwMS who did not achieve NEDA, *p* < 0.001. The mean time to EDSS 6 in pwMS who achieved MEDA at year one was 34.1 years (95% CI 31.3–36.8) vs. 29.1 years (95% CI 23.1–35.1) in pwMS who did not achieve MEDA (*p* < 0.001). If rebaselining, mean time to EDSS 6 was 44.5 years (95% CI 40.4–48.5) in pwMS achieving NEDA vs. 29.6 years (24.2–35.0) in pwMS who did not achieve NEDA, *p* < 0.001, see Figure 5. The hazard ratio of reaching EDSS 6 in the NEDA vs. no NEDA groups at year one and at rebaseline was significant in the cox regression analysis. The effect remained strong after adjusting for gender, age at diagnosis, time from onset to diagnosis, type of DMT and time of DMT initiation, but was no longer significant, see Table 4.

**Table 4:** Hazard ratio of reaching EDSS 6 in those who did and did not achieve NEDA at year one and at rebaseline, before and after adjustment using cox regression analysis.

	**HR of reaching EDSS 6**	**95% CI**		***p***
NEDA year 1, crude	0.349	0.189	0.644	< 0.001
NEDA year 1, adjusted	0.487	0.161	1.478	0.202
Female	1.252	0.493	3.176	0.636
Age at diagnosis	1.062	1.013	1.114	0.012
High efficacy DMT first	0.524	0.177	1.550	0.243
Years from onset to diagnosis	0.686	0.549	0.857	< 0.001
DMT within 6 months of diagnosis	1.125	0.414	3.060	0.817
NEDA rebaseline, crude	0.385	0.218	0.678	< 0.001
NEDA year 1, adjusted	0.421	0.164	1.085	0.073
Female	1.421	0.560	3.606	0.459
Age at diagnosis	1.070	1.020	1.122	0.005
High efficacy DMT first	0.450	0.146	1.385	0.164
Years from onset to diagnosis	0.668	0.520	0.856	0.001
DMT within 6 months of diagnosis	1.539	0.525	4.507	0.432

HR, hazard ratio; NEDA, no evidence of disease activity; DMT, disease modifying therapy; CI, confidence interval.

### What is NEDA fail?

**Figure 6:**
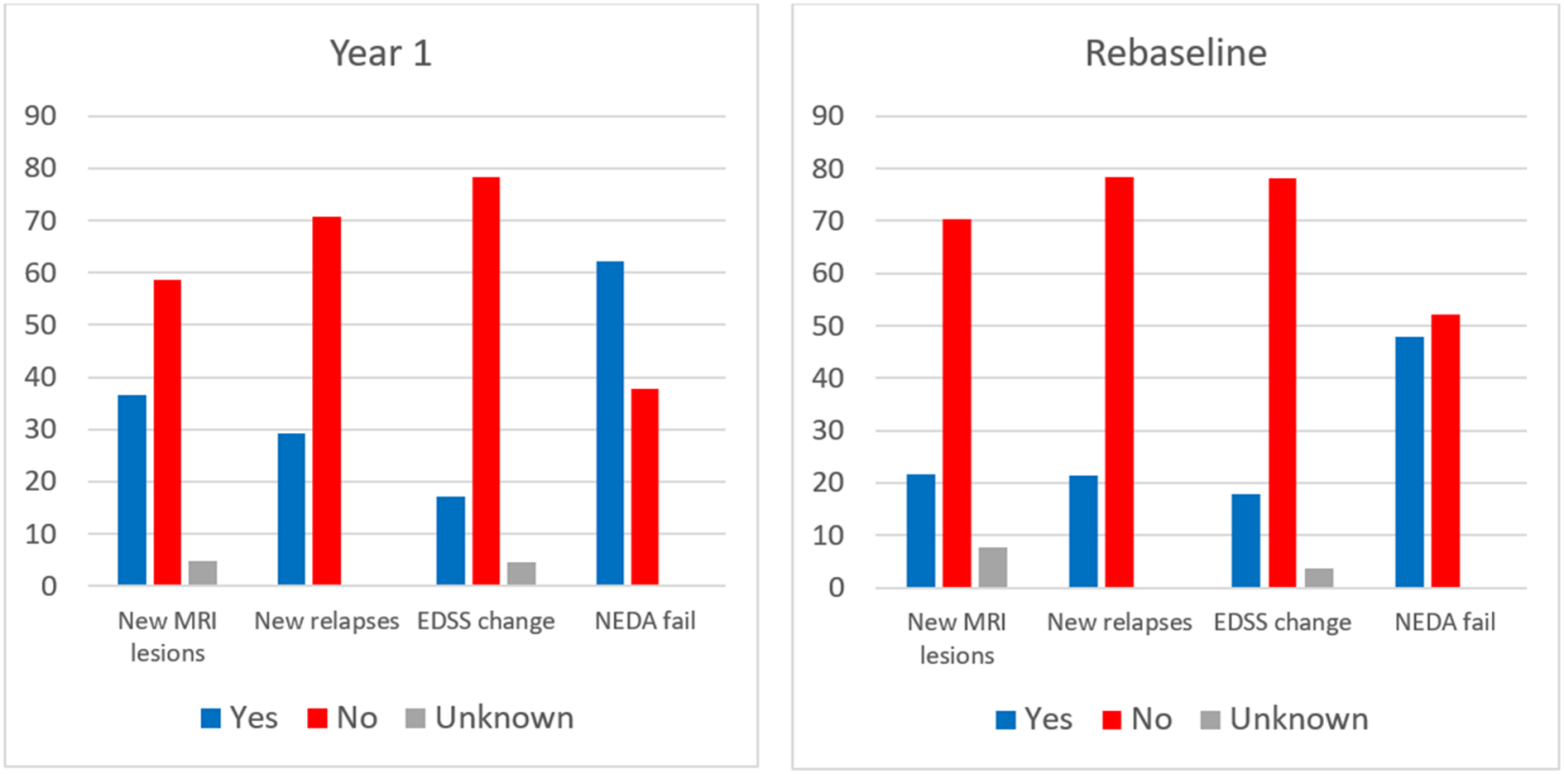
Individual components of NEDA in all pwMS in year one and at rebaseline. EDSS expanded disability status scale. NEDA no evidence of disease activity. MRI magnetic resonance imaging.

Figure 6 shows which components are responsible for NEDA fail in all pwMS combined and in pwMS with NEDA fails only (*n* = 334 at year one and *n* = 213 when rebaselined). In the NEDA fails at year one, 70% had one failed components, 28% had two failed components and 2% had three failed components of NEDA. New MRI lesions are the most common cause of NEDA fail, followed by new relapses. Of pwMS with one failed components, 49% achieved NEDA when rebaselining, while 38% of pwMS with two failed components and 14% of pwMS with three failed components achieved NEDA when rebaselining.

## Discussion

In this Norwegian population-based, real-world study, we found that NEDA status at rebaseline can predict the long-term disease course in pwMS. Starting a high efficacy DMT is associated with longer time to NEDA failure than starting moderate therapies.

Other studies have shown the importance of achieving NEDA in predicting long term disability. Rotstein et al. found that NEDA at 2 years had a positive predictive value of 78.3% for no progression at 7 years (4). Equally, long-term follow-up data from the randomized trial of interferon beta-1b showed that pwMS who experienced clinical NEDA for the two-year period following inclusion were less likely to develop negative disability outcomes after 16 years (5). Rio et al. showed that pwMS treated with interferon-β who had achieved NEDA at 1 year remained free of disease activity in the long-term (6). Limited disease activity early in the disease course is widely regarded as a good prognostic sign since most inflammatory-associated damage occurs early (16, 19). Consequently, early treatment with high efficacy therapy is important to prevent irreversible disability (20–22). Although there was no increase over time in the proportion of pwMS achieving NEDA in year one, there was an increase over time in the proportion of pwMS achieving NEDA when rebaselining, suggesting we are getting better at stabilizing the disease early.

Another important finding from the present study is that pwMS with NEDA from rebaseline after one year have a longer time to EDSS 6 than pwMS with NEDA from time of diagnosis. Disease modifying therapies do not reach full clinical efficacy until after several weeks to months, and NEDA must be adapted accordingly when utilized as an outcome measure. While 51% of the trial participants treated with natalizumab reached NEDA after 2 years in the original randomized control trial, this rose to 71% when the trial participants were rebaselined at 12 months (23). In our study, time to NEDA fail and EDSS 6 increased when rebaselining the population 1 year after diagnosis. Conversely, Tsantes et al. found that a fixed 6 month-rebaseline only had a small impact in improving NEDA prognostic value at five and seven years compared to baseline, though their study population was smaller than ours and their outcomes differed (7). It is also worth noting that the hazard ratio of reaching EDSS 6 at year one and rebaseline is significantly associated with early diagnosis and moderate efficacy therapy as a first therapy. This again supports the importance of early diagnosis and high efficacy therapy early in the disease course to prevent long-term disability.

However, whether NEDA is a good tool to predict long term disability is controversial. Several authors have argued that failure to achieve NEDA is not necessarily a good predictor of long-term disability (9, 10). Prosperini et al. (8) showed that NEDA does not ensure long-term clinical stability because disability accrual may occur as both relapse associated worsening (RAW) and progression independent of relapse activity (PIRA) in a proportion of pwMS. In fact, NEDA is by no means a perfect tool. For one, the clinical measures are crude and the concept is overly reliant on MRI findings, with the main focus on focal inflammatory disease activity, thus underestimating silent neurodegeneration and chronic lesions (24, 25). White matter microstructural deterioration occurs even in pwMS with NEDA (12). Subclinical relapses, atrophy and disease progression may still occur without clear clinical relapses. In addition, the EDSS is inadequate when measuring upper limb and cognitive functions (26). Even Prosperini et al. found that three in four pwMS were correctly classified on the basis of NEDA-3 (8), suggesting that NEDA early in the disease course is a good foundation to build future prognostic tools. Adding brain volume loss or atrophy has been proposed as NEDA-4 (13) and NEDA-5 may in the future also include cognitive tests, neurofilaments and pwMS reported outcome measures (11).

Finally, most pwMS only fail one component and new MRI lesions are the most likely cause of NEDA failure. Surprisingly, there was a significant difference between the two hospitals in achieving NEDA with MRI findings representing the main discrepancy. This serves as a reminder that all the three components of NEDA are highly subjective and there is no real consensus regarding the definitions of the different components of NEDA (11, 27). Apart from between-rater variability (28), MRI findings may vary across centers due to differing MRI machines and protocols (29). Relapses are subjective, both for the pwMS and the clinician, and dependent on pwMS -clinician contact (30). EDSS is also subject to significant between-rater variability (31). The general population components and socioeconomic differences between the two counties, such as smoking, education, employment and access to health care, may also affect the outcome (32). All this highlights the importance of complete populations and the hazards of comparing different populations (1).

Our study was population based with a geographically well-defined sample, which minimizes the chance of selection factors adversely affecting the study results. Real-world studies, such as this, are not restricted by stringent inclusion criteria but instead assess the entire heterogeneous population and can therefore be generalized beyond their study frames (33). Another benefit to this study is its contemporary population. Both studies supporting NEDA and studies opposing the use of NEDA as a predictor of long-term disability are based on older pwMS treated primarily with injection therapies. All Norwegian MS neurologists had complete access to all therapies available in Europe at the time of approval, and all drugs were reimbursed.

However, real-world data is subject to missing data, which is a source of potential information bias and selection bias. We did not have sufficient NEDA data on 13% of the complete population. The population without information about the outcome were more likely to have progressive disease at onset, they were older and time from onset to diagnosis was longer. It is likely that adding this population would shorten the time to NEDA fail, though it is noteworthy that time to EDSS 6 did not differ between the pwMS included and those missing. In addition, we were missing one component of NEDA in 9% of pwMS at year one, all of which had a positive finding in one of the two remaining components. However, we did repeat the analyses using only pwMS with complete information on all three components of NEDA (*n* = 488, data not shown), and there were no noteworthy differences between the groups. Another potential bias is observation bias. PwMS on natalizumab are seen monthly, and are more inclined to mention relapses to their treating MS team than pwMS seen less often (30). There is also a lower threshold for taking MRIs if there are new symptoms. In addition, in the very beginning of the study period, MRIs were done more routinely on pwMS on high efficacy therapies, thus allowing for more MRI lesions in these pwMS. Despite this, we found a significant beneficial difference in time to NEDA fail in pwMS of high efficacy therapies.

## Conclusions

NEDA-3 from rebaseline after 1 year, once treatment is stabilized, can predict the long-term disease course in MS. Starting a high efficacy DMT is associated with longer time to NEDA failure than moderate therapies. Finally, most pwMS only fail one component and new MRI lesions are the most likely cause of NEDA failure.

## Data availability statement

The datasets presented in this article are not readily available because the Regional Ethics Committee in Norway (REK 2015/670) approved this study under the condition that strict privacy concerns were respected, and that data was not made publicly available. Requests to access the datasets should be directed to CS, cecsim@vestreviken.no.

## Ethics statement

The studies involving human participants were reviewed and approved by Regional Etisk Kommite. Written informed consent for participation was not required for this study in accordance with the national legislation and the institutional requirements.

## Author contributions

CS: design and conceptualization, major role in the acquisition of data, analyzed and interpreted the data, and drafted the manuscript for intellectual content. HF: design and conceptualization, major role in the acquisition of data, interpreted the data, and revised the manuscript for intellectual content. LB: design and conceptualization, major role in the acquisition of data, and interpreted the data. KB: design and conceptualisation, interpreted data and revised the manuscript for intellectual content. CB: analyzed and interpreted the data and revised the manuscript for intellectual content. PB-H: design and conceptualization, interpreted the data, and revised the manuscript for intellectual content. EC: design and conceptualization, interpreted the data, revised the manuscript for intellectual content, supervision, and project administration. All authors contributed to the article and approved the submitted version.

## Funding

The study was funded by an unrestricted research grant from Sanofi (Grant number: GZ-2014-11451). The funder was not involved in the study design, collection, analysis, interpretation of data, the writing of this article or the decision to submit it for publication.

## Conflict of interest

Author CS has received personal compensation for lectures and/or serving on scientific advisory boards from Sanofi, Merck, Novartis, BMS, and Biogen Idec and received unrestricted research grants from Sanofi and Novartis. Author HF has received unrestricted research grants from Biogen Idec and Novartis. Author KB has received personal compensation for lectures and/or serving on advisory boards from Biogen, Novartis, and Merck. Author LB has received unrestricted research grants from Sanofi, and advisory board honoraria from Sanofi, Merck, and Biogen. Author PB-H has received advisory board and/or speaker honoraria from Novartis, UCB, Teva, Merck, and Biogen Idec. Author EC has received personal compensation for lectures and / or serving on scientific advisory boards for Almirall, Biogen, BMS, Janssen, Genzyme, Merck, Novartis, Roche, and Teva. Her department has received unrestricted research grants from Biogen, Novartis, Merck, and Genzyme. The remaining author declares that the research was conducted in the absence of any commercial or financial relationships that could be construed as a potential conflict of interest.

## Publisher's note

All claims expressed in this article are solely those of the authors and do not necessarily represent those of their affiliated organizations, or those of the publisher, the editors and the reviewers. Any product that may be evaluated in this article, or claim that may be made by its manufacturer, is not guaranteed or endorsed by the publisher.
